# Recruitment and retention into longitudinal health research from an adolescent perspective: a qualitative study

**DOI:** 10.1186/s12874-022-01802-7

**Published:** 2023-01-16

**Authors:** Stephanie T. Jong, Rebecca Stevenson, Eleanor M. Winpenny, Kirsten Corder, Esther M. F. van Sluijs

**Affiliations:** 1 UKCRC Centre for Diet and Activity Research (CEDAR) at the MRC Epidemiology Unit, University of Cambridge School of Clinical Medicine, Institute of Metabolic Science, Cambridge Biomedical Campus, Box 285, Cambridge, United Kingdom; 2 School of Health Sciences, University of East Anglia, Norwich, United Kingdom

**Keywords:** Adolescent, Early adulthood, Recruitment, Retention, Study participation, Longitudinal, Transition

## Abstract

**Background:**

High quality longitudinal studies investigating changes in health behaviours over the transition into early adulthood are critical. However, recruiting and retaining adolescents is challenging. This study explored adolescents’ perspectives of signing up to and continuing involvement in a hypothetical longitudinal health research study.

**Methods:**

Forty-eight individuals (15-20y) participated in nine in-person focus groups about recruitment and retention in research. Participants were (a) school students in the last year of compulsory school (Year 11, 15-16y), (b) school/college students in Sixth Form (Year 13, 17-18y), (c) Further Education students studying after secondary education, but not higher education (16-18y) and (d) young adults not in education, employment, or training (18-20y) across England. Thematic analysis resulted in seven themes.

**Results:**

Driving factors for sign-up included social connection e.g., joining with peer groups, personalised feedback, and incentives, primarily financial. Key barriers were lack of interest, the perception of commitment, and timing of recruitment. Young people preferred recruitment processes via social media with messages tailored to their motivations, monthly data collection of maximally 20–30 min, and hybrid data collection with some in-person contact with a consistent, non-judgemental researcher. The provision of autonomy, choice, and financial incentives were perceived to promote retention.

**Conclusions:**

Adolescent recruitment and retention strategies need to align with contemporary interests and motivations. Studies should involve adolescents early to develop a planned, systematic approach to participant sign-up and follow-up. Effective and ineffective recruitment and retention strategies should be reported as part of study findings. Future research should trial how perceived barriers to study engagement can be overcome.

**Supplementary Information:**

The online version contains supplementary material available at 10.1186/s12874-022-01802-7.

## Background

The transition from adolescence into early adulthood is a distinct period of development, characterised by instability and identity exploration [1]. During this transitionary period, important changes in the physical and social environment and lifestyle take place, which influence many health determinants [2, 3]. Additionally, the changes in this period of development mean young adults are more susceptible to risky health behaviours [4], but also provide a unique opportunity to study and implement behaviour change programmes [5, 6]. Longitudinal health studies allow researchers to understand developmental changes, and provide insight as to how health behaviours and outcomes change with age [7]. There has been growing interest in studying risk factors and health conditions over the life course, linked to the long-term associations with adult disease [8]. For example, physical inactivity in adolescence has been associated with adulthood inactivity [9–11], which is linked to increased risk of diabetes, cancer and mortality [12, 13]. However, the success of longitudinal research is dependent on effective recruitment and minimizing participant attrition [14], two factors which have been identified as the most challenging aspects of adolescent research [15].

Previous research has indicated that effective adolescent recruitment strategies include taking time to develop relationships with partners and the community, providing information sessions and clear messages regarding the study, obtaining support from key stakeholders, creating a study identity, and using a correct and optimal consent process [16]. Reflections from public health researchers working with adolescents align with these results, affirming three recommendations for effective recruitment of adolescents: 1) collaborating with gatekeepers (one who controls access to participants), 2) using additional recruitment tools (e.g., flyers, informational sheets, email, face-to-face interaction, radio etc.), and 3) understanding your target population [17]. Other studies have described positive experiences resulting from personal contact with parents/guardians and close cooperation between school personnel and study staff [18], electronic methods, flyers, word of mouth, and respondent-driven sampling procedures [15], contacting former participants and attendees of the research centre’s outreach events, as well as social media and word-of-mouth [19].

In terms of retention, successful strategies include fostering a positive relationship and open lines of communication, maintaining consistency of the contact person, a person with good interpersonal skills, collecting multiple types of participant contact information [7, 20], expressing appreciation for participant's time and effort, allowing time for peer socialization [21], and using incentives [7, 18]. Using contests were not found useful [7]. Interestingly, in their systematic review and meta-analysis of retention strategies in longitudinal cohort studies, Teague et al., [22] concluded that a greater number of retention strategies might not be associated with improved retention. Instead, strategies identified as most effective at maximising cohort retention were barrier-reduction strategies i.e., those that reduced participant burden (e.g., flexibility in data collection methods).

More recently, understanding participant motivation has become a priority for health research. In identifying and prioritizing unanswered questions and uncertainties around trial retention, 456 respondents (key stakeholders involved in randomised trials such as staff, researchers, and patients/public) of the PRioRiTY II (Prioritizing Retention in Randomised Trials) project [23] ranked ‘What motivates a participant’s decision to complete a clinical trial?’ as number one. Stakeholders believed that future research on improvements to retention should focus primarily on individual motivation to complete trials. Taking a theoretical approach, according to Self-Determination Theory (SDT), intrinsic motivations come from within and describe how performing an activity can be motivated out of curiosity or inherent interest [24]. That is, the activity is not a means to gain or avoid something but is an end in itself. Conversely, extrinsic motivations are described as an outside force that encourages someone to undertake an activity as it leads to a separable outcome [24]. This outcome could be positive, such as payment, or negative, such as avoiding punishment. Understanding motivations for participation is a key factor in recruitment and retention of participants.

Qualitative research with young people into recruitment and retention has been relatively limited. Results from one study with 13–17-year-old African American adolescents with a chronic condition (*n* = 15) found that they desire honest communication, opportunities for building relationships with other teens, incentives, and choice during the intervention [25]. In a mixed methods study with 18–25-year-olds focussing on improving recruitment in weight loss programs, participants preferred online recruitment with tailored messages specific to their motivations and preferences of the age group, low intensity, and brief data collection measures with some in-person contact, autonomy and choice, and a fiscal incentive [21]. In a qualitative study with a similar age group (18 + years), four main themes emerged: transportation, incentives and motivation, caregiver concerns, and the social and physical environment considerations [26].

Much of the previous research has focussed on participants involved in longitudinal studies, as well as specific sub-populations such as young people who smoke [27], adolescents with abuse-related posttraumatic stress disorder [20], outpatients [26], and females [28, 29], rather than recruiting and retaining at a population-level. There is a paucity of qualitative research and with a contemporary adolescent population. Research with participants at different points of transition in their lives, for example those already transitioned out of secondary school or participants not in education, employment, or training (NEET), is limited. Given the importance of following adolescents into adulthood, researchers need to understand their target population further to develop meaningful, contemporary, reliable designs that maximize recruitment and retention of adolescents in research [17]. The aim of the current study was to explore the perspectives of adolescents and young adults on participation and continued involvement in population-based health research (see, for example, the Adolescent Health Study [30]) to understand factors that may improve recruitment and retention for contemporary, longitudinal research in this population.

## Methods

### Sample and study sites

We aimed to recruit older adolescents and young adults between the ages of 16 and 30 years. Participants were members of the general population and were not recruited based on current participation in a study or based on any health condition or behaviour. In order to hear a range of experiences, those with different levels of education and those NEET were included in the study. Participants were recruited between October 2018 and March 2019 from four subgroups: (a) Year 11 (when adolescents are 15-16y), (b) Sixth Form (Year 13, 17-18y), (c) from any level at a Further Education (FE) college (any study after secondary education that is not part of higher education [31]; 15-18y), and (d) adolescents/young adults NEET (from age 17y). A mix of purposive and convenience sampling was used.

### Procedure

Schools/colleges in East Midlands were approached to participate in the study. These sites were identified linked to the location of the researcher (RS) conducting the focus groups. Head teachers or Principals of the secondary school or FE college were provided an information sheet and were invited to provide a setting for recruitment and to host the focus groups. Once agreed, the school/college identified students from their school/college to invite to participate. The school/college were provided with invitation packs, including an information sheet, to disseminate to students. Schools were advised that we were seeking participants from specific year groups, with a mix of ability levels and subjects. Participants from the FE college were recruited from different subjects across two geographically dispersed sites. Interested students were able to express their interest in taking part by contacting the study team by email or phone. Schools/colleges provided time out of lessons to participate in the focus groups.

For young adults NEET, initially, the local council, local employers and charitable organisations working with young adults NEET were approached via email to facilitate recruitment for the study. This did not result in successful recruitment. A young adult, who identified as NEET, was approached though a personal contact of the researcher (RS) and consented to participate. Snowball sampling was then undertaken to recruit a further three participants. Participants were not known to the researcher.

All participants provided written informed consent. Participants were encouraged to discuss study participation with their parents/care givers, but provided written consent themselves in line with the United Kingdom Human Research Authorities [32]. Participants were provided a £10 gift voucher in appreciation of their time, and signed confirmation of receipt of the voucher. The consent procedure regarding the inclusion of minors of the study, and research design and procedures were reviewed and approved by the School of Humanities and Social Sciences Research Ethics Committee of the University of Cambridge 18/175. The study was performed in accordance with the Declaration of Helsinki [33].

### Data collection

Participants completed a brief questionnaire to obtain sociodemographic information before the start of the focus group. This included questions about age, ethnicity, gender, current living circumstances, parental education and employment, employment/education status, free school meal status and the Family Affluence Scale [34, 35].

Two researchers independently conducted focus groups (RS and SA). All focus groups were audio recorded. Structured focus groups were identified as a suitable data collection technique, given their ability to encourage participants’ views and perceptions. Focus groups were conducted with peers in settings already familiar to participants. This has been suggested as useful in increasing participant comfort [36], but also gives them a chance to identify and clarify their own views by considering and debating ideas with others known to them [37]. This interactivity has the potential to lend itself to increased depth of dialogue [38]. Additionally, the researchers also considered that this format would help facilitate a rapport with the researcher leading the focus groups as it was assumed that this would be the first involvement with research for many participants.

A topic guide (Supplementary file 1) was created based on a brief review of the literature and based on the research team’s experiences of conducting longitudinal studies with this age group. The key focus of the topic guide was to investigate how to recruit and retain adolescents and young adults in longitudinal cohort studies. Given the background and research interests of the research team, questions were also asked around population-based health research, which included questions around recruitment to health-related studies, and physiological health measures. The topic guide acted as a means through which other questions were asked, particularly in terms of clarification. The topic guide covered the following topics: recruitment, practicalities of data collection, keeping participants engaged and motivated over prolonged periods of time, and research team contact during the transitional period of adolescents into early adulthood. Ideas generated from focus groups helped to develop the prompts used in subsequent data collection.

All focus group participants were asked to base their thoughts as if they were considering participating in a hypothetical longitudinal study. When describing a hypothetical longitudinal study, the interviewer prompted consideration for varying lengths of time, including ‘six months’, and using words such as ‘long-term’, ‘a year’, ‘two years’ and beyond. The interviewer also described data collection measures that could be used in the hypothetical study; for example, tracking steps via a pedometer, and questionnaires. Further interviewer questions after participant responses also prompted consideration from a longer-term basis. For example, when discussing data collection methods, the interviewer asked, ‘Would you mind doing that for a year?’ Regarding recruitment-based questions, participants were further prompted to consider their responses as if they were considering participating in a health-based hypothetical study. We did not ask participants whether they had a specific health condition.

### Data analysis

All focus groups were transcribed verbatim and anonymised. Focus group data was analysed using codebook thematic analysis [39]. Two researchers followed Braun and Clarke’s six stages of thematic analysis [40], interweaving elements of codebook thematic analysis. Both researchers independently organised the data into interesting and meaningful collections of initial codes. The researchers used NVivo [41] to facilitate the data analysis process. Coding was both inductive and deductive. After initial coding, the researchers met to discuss their analysis, and to clarify any differences in interpretation. Once the transcripts were coded completely, the codes were interpreted into broader themes; codes were aligned to recruitment and/or retention topics. In this way, some of the themes were determined in advance of full analysis. The researchers met to review and define themes with the broader research team. The aim of this iterative process was to act as ‘critical friends’ in discussing agreements and disagreements to promote reflexivity.

## Results

### Study sample

Twenty educational sites were contacted via email, and two responded: one secondary school, and one FE College in the East Midlands of England. Young adults NEET were recruited from the North West and included a mixture of individuals not in employment or training, as well as some who had part-time employment.

We recruited 48 individuals, aged 15–20 years. Nine focus groups were conducted from October 2018 through to March 2019 and were hosted on the school/college site or within the local community (e.g., the town library) for those not in education. The focus groups lasted between 21 and 35 minutes. Participant numbers varied across focus groups (*n* = 4–7). Participants were (a) school students in the last year of compulsory school (Year 11, 15-16y; *n* = 13), (b) school/college students in Sixth Form (Year 13, 17-18y; *n* = 16), (c) FE students (16-18y; *n* = 11) and (d) young adults NEET, who ideally did not have any post-school qualifications (18-20y; *n* = 4). Table 1 present demographic characteristics of the participants.

**Table 1:** Demographic characteristics of focus group participants^a^

Variable	Category	Year 11 (*N* = 13)	Sixth form (*N* = 16)	Further Education College (*N* = 11)	NEET (*N* = 4)	Total (*N* = 44)
**Age**	15-16y	13 (100%)		3 (27%)		16 (36%)
	17-18y		15 (100%)	8 (73%)	1 (25%)	24 (55%)
	19-20y				3 (75%)	3 (7%)
**Gender**	Female	8 (62%)	14 (88%)	7 (64%)	2 (50%)	31 (70%)
	Male	5 (38%)	2 (12%)	4 (36%)	2 (50%)	13 (30%)
**Ethnicity**	White	12 (100%)	5 (31%)	10 (91%)	4 (100%)	31 (72%)
	Asian		8 (50%)			8 (19%)
	Other		3 (19%)	1 (9%)		4 (9%)
**Family affluence score**	Low	1 (8%)	0	1 (9%)	2 (50%)	4 (9%)
	Medium	4 (31%)	6 (38%)	3 (27%)	1 (25%)	14 (32%)
	High	8 (62%)	10 (73%)	7 (64%)	1 (25%)	26 (59%)
**Free school meals**	No	12 (100%)	11 (73%)	1 (9%)	2 (50%)	26 (62%)
	Yes		4 (27%)	10 (91%)	2 (50%)	16 (38%)

^a^Percentages calculated from available data. Missing data: Full questionnaire: *n* = 4 (all NEET); Ethnicity *n *= 1; Free School Meals *n* = 2; Age *n* = 1

### Themes

Analysis determined seven themes illustrated in the below section: 1) preferred modes of recruitment, 2) requirements of a recruitment advertisement, 3) interest and relevance of the research to me, 4) incentivising participation, 5) commitment over long transitioning periods of time, 6) user friendly data collection, and 7) maintaining contact throughout the study. Most themes were conceptualized as domain summaries, reporting what participants said in relation to the interview question [39] (deductive); for example, requirements of a recruitment advertisement, and preferred modes of recruitment. Other themes were shared meaning-based patterns (inductive), for example, commitment over long transitioning periods. Illustrative anonymised quotes typify the data from the focus groups. Few differences were observed between adolescent and young adult respondents and therefore findings are combined unless specifically stated.

#### Preferred modes of recruitment

Participants specified several recruitment modes that they perceived would be effective. Social media was mentioned across all focus groups. Instagram was the preferred method of recruitment. Specifically, participants advocated for Instagram advertisements, and the ‘swipe up’ feature on the app, which allows those who are interested to find out more about a particular product/event/study etc. Facebook was the second most popular social media recruitment method mentioned, however, this was not endorsed by Sixth Formers. Snapchat and Spotify were also mentioned in the discussions about recruitment methods.

Participants in Year 11, Sixth Formers and those in FE, noted school/college email circulations as an additional method of recruitment. These were detailed as an effective recruitment method, currently used to circulate job vacancies and voluntary positions. Additional suggestions included posters or having the research team come into classes or assemblies.

An additional key element of recruitment mentioned by participants from Year 11, Sixth Formers, and FE students, was the option to participate in research alongside peers from their friendship groups. Participants stated that participating in the research process was something they would rather do with friends, as opposed to individually. They expressed that if friends were involved in the research with you, that it might help you to become involved and maintain involvement. Some participants stated that they would avoid focus groups with people they did not know as it would make participants ‘feel weird’ and ‘refrain from saying stuff’ (Sixth Form participants). A Year 11 student commented:

I’d be a lot more comfortable if there’s people that I knew doing it as well, so if you could reach out to like more of a group instead of just individual people from everywhere, I think that’d be better.

However, participants NEET were more open to meeting new people as this was identified as more difficult after they left education.

#### Requirements of a recruitment advertisement

Participants indicated that they wanted to know from the initial advertisement what the study was about, a rationale of why the research is being conducted, what they would get out of participating, how and where data collection would take place, the requirements of them, what the research will do, and how it will benefit others. Sixth Formers also wanted to know where the research would be published. A few participants suggested that they were more likely to get involved in a longitudinal study if they knew what their participation contributed to on a broader scale (e.g., how it would help in the future).

In describing the requirements of how a recruitment advertisement should look, participants prioritized visibility of the institutional logo on the initial advertisement of the study. All groups suggested that sign up would increase if the study was conducted by a ‘trusted’ and ‘credible’ institution. Participants suggested this should be made clear by the branding used and that a study website should have a secure lock following the URL. In addition, participants wanted the advert to ‘look interesting’ and be ‘visually appealing for that age group’. For participants not in education, this looked like the inclusion of imaging, or positive facts/statistics. Sixth Formers on the other hand, suggested a catchy tagline, with the advert showing something that they could relate to, something to do with their life, so that they could imagine people their age participating. It was suggested that including images of people older than them would cause hesitancy in participation. All focus groups reiterated the importance of simple messaging use on recruitment advertisements.

#### Interest and relevance of research to me

There was a firm belief from participants across all focus groups that sign up required interest in the topic area of the study. Involvement was described as purely dependent on interest. In a discussion with Sixth Formers, a hypothetical study of ‘lifestyle behaviours, diet, exercise and health’, was suggested. While most expressed an interest, one participant said that the title alone would not appeal to them. Further Education students similarly discussed that some topics would turn people off. For example, one participant mentioned that a study about fitness or physical activity would not appeal to them. If there was no interest in the area, then it was believed that people would not volunteer to participate:

I think it’s more specific to the person, if you’ve got the interest then you’re going to want to take part, and if you’re not interested in the slightest then you’re going to be like it’s not worth my time, even with the incentives, you’re really, really not interested in whatever, it’s not going to be anything for you.

(NEET focus group).

Interest promoted involvement and was further linked to the time commitment of a longitudinal study. If participants were interested, they were happier to commit time to the study. One way of making a study interesting was variability in measurements over periods of time. For example, one Sixth Former participant stated:

If you said it in like an exciting way, like instead of saying “oh you’re going to be in our study for ten years”, like this makes me feel “Oh, that’s long”. But if it was like an engagement that we do different things and stuff, then it just makes it more interesting.

#### What’s in it for me? Incentivising participation

All focus groups included discussions around perceived personal benefit. The following examples were given: motivation, cooking skills, learning, or, specifically for Sixth Formers, enhancing their personal statement. Other discussions had a focus on the receipt of personalised feedback as a personal benefit to facilitate retention. For example, up-to-date personalised feedback about their overall health, their individual steps per day, or weight loss. In turn, this personalised feedback would potentially facilitate actions on how they could improve their health.

Incentives were an important identified factor for recruitment and retention. For participants, incentives were an assumed part of the research process:

P-1:I wouldn’t do it for nothing. I’ll just say that now. You’ve got to be rewarded for doing it

P-3:You need something to actually make you want to do it. And like all studies do give people money, no-one really does it without

(NEET focus group).

Participants suggested incentives including money, a monetary voucher, a subscription (e.g., Netflix), discount codes for favourite stores, or, for younger participants (Year 11), a trip to Alton Towers (an amusement park), or music festival tickets (Sixth Formers). Financial incentives were discussed as being dependent upon what was required of the participant. For example, some participants suggested £10 for an hour. Participants indicated that each measurement session or data collection measure should equate to an incentive. This could be provided once a month, rather than each time a participant completed a survey, and could also be accumulative over a time e.g., you completed three out of the five surveys and will receive X amount. All participants preferred to receive a guaranteed incentive, rather than a chance to win a larger financial reward.

An important factor for some participants was that they had the choice or options to select from. Participants suggested that through data collection, e.g., focus groups, the research team could build a relationship with participants to offer an incentive that they would like.

An additional but subsequent thought was given to how participants’ involvement in the research would help others:

I think if you know the data’s going to be helping others as well, that would like be interesting.

(Sixth Form focus group).

Some participants detailed that they would want to be involved in a larger scale study to have a ‘grander’ impact, understanding their role in ‘what is going on in the bigger picture’.

#### Commitment over long transitioning periods of time

Commitment was a key factor in the decisions on whether to become involved in a study. Before committing to a study, participants wanted to be clear on timeframe:

When and stuff like that, because if it’s like a study for our age, like we’re doing a lot so it’s like yeah like a timeframe, but probably be suitable to know like when we’re meeting, when you can, when you can’t, like that.

(Sixth Form focus group).

Discussions around the time investment required differed across participant groups. For Sixth Formers, a ‘long time’ was identified as ‘a couple of weeks’ by one participant, ‘a couple of months’ by another, and ‘about a year’ by another participant. Further Education participants indicated that three years was a ‘proper commitment’. For recruitment into studies, participants in Sixth Form grappled with the time investment:

“If it takes a lot of your time then I wouldn’t do it, but if like all the commitment you have to make are fairly short then yeah.”

Participants NEET also discussed commitment in relation to time investments:

P-1:I’d just be like would I be able to commit to it? Because *[sighs]* I don’t know, it would be hard to.

P-4:At the same time, I feel like committing to it would help with time management and coming to things in future as well, so I feel like I would

A vital consideration linked to recruitment and retention was timing. All focus group discussions acknowledged the transitioning time of adolescents to early adulthood. Some Sixth Formers discussed that if their involvement commenced at 16 years of age, and the study was for two years, that they would be involved as they knew they would be at college for this time. However, after this time, participants indicated that their involvement would require a greater commitment as it would be alongside university, work, and travel plans. Young adults NEET agreed with this idea and identified that after school, general life, prior obligations, social events, work, and the idea of not knowing where they would be in a years’ time were key barriers to committing to a longitudinal study:

“It’s like can you like maintain that commitment while everything else is changing around you as well?”

Despite the perceived ease of participating while at college, Sixth Former participants also discussed barriers to their involvement. One consideration included the academic pressure at college:

I think just like it requires commitment and then you just like can’t be bothered because then you’ve got like your A-levels and that work to do.

Further Education participants also indicated that school/college would take precedence, and could potentially override any recruitment during this time.

Due to academic commitments, participants said that they may not reply during this time, and that it was important for the research team to avoid communications during busy periods. Some indicated that they would be happier to participate if it did not ‘affect your academic work’, and that key transitional points were to be avoided in general.

Participants gave thought as to how to advertise longitudinal studies to avoid immediately deterring potential participants. Ten years was deemed a long time, however, one Sixth Form participant stated that it would be better advertised as how many times you would be required to meet face-to-face as ‘that seemed less’. Participants detailed that they would want ownership over the length of their involvement from the beginning of the study. One FE participant provided an example:

If you do it, it’s like you do a year and then you have a possibility of a second year but then what you get for the second year is better than the first year, like loyalty kind of thing.

#### User friendly data collection

Participant discussions around data collection emphasized flexibility and convenience. Participants preferred local data collection locations, those close to work or study, to ensure that the site was accessible and familiar to them. Suggestions included school/college sites, a local hospital or general practice, local library or somewhere central to the area. Data collection at home was considered ‘weird’ by participants in Year 11.

Data collection needed to be flexible and work around their timetable. Some preferred drop-in sessions to allow for flexibility and change of plans, while others preferred a booking system to ensure a specific time. Time commitment suggestions varied. Generally, participants indicated that they would be happy to complete data collection procedures once a month, for 20–30 min per time. Many preferred one data collection point for all measures ‘to get it all over and done with in one moment in time’ (Year 11 focus group), rather than completing a daily survey; those in Year 11 stated that they did not have the ‘motivation’ to do this.

Discussions from various focus groups stated the importance of wording choice for data collection. Simple messages were key to effective communication. Participants in Year 11 suggested that there was a need for simple questions. A participant in FE College also commented on the importance of wording choice:

…explain the actual word ‘focus group’ because when she (teacher) told us to come here (to attend the focus group) we were like we’ve got to talk about our feelings and stuff.

Most of the participants preferred an app for online survey data collection. An app would need to be easy to use, have no cost, and did not require much phone storage (megabytes). An additional stipulation was that the app would not track GPS. This was described as ‘creepy’ and ‘intrusive’. The alternative, having to post the survey off, was considered additional effort and a barrier for some.

Some Year 11 participants preferred to complete a survey for data collection whilst at school. This was due to perceived ease:

“Because I feel like I’d lose it, or I wouldn’t take it seriously, if it’s at school in the classroom, get it done, then give it back straightaway.”

For these participants, being at school changed their mind-set of participating; the study became linked to schoolwork. Completing a survey at home would feel like homework and would encroach on their leisure time. This was reiterated by a Sixth Form focus group where data collection was preferred to be embedded in college time as outside of college hours ‘seems like extra effort’. This was contrasted by participants not in education who preferred data collection after work and not on weekends, as ‘weekends is my time’.

All participants indicated that they would be happy to wear a device, such as a physical activity monitor. Participants wanted to know what the device was measuring and stated that the device must be small and comfortable, easy to hide for social occasions, and not compromise their work or involvement in sport. The preference was a watch monitor, similar to a Fitbit, however, this was not seen as viable for sports such as netball, and a belt may be preferred in this situation.

#### Maintaining contact throughout the study

All participants stated that they would be happy to provide their phone number and email address for maintaining contact; two ways to be contacted was deemed appropriate by participants. Other suggestions included home address (Year 11) and Facebook (NEET). Many indicated that they did not check their emails regularly, and that they would prefer tailored and specific text communication via their phone, rather than a phone call.

If the study did have an app, notifications were stated as the best way to keep in touch, though minimal notifications were preferred. Some indicated they may delete the app if notifications were too frequent. To avoid overburden, a similar preference was expressed for other forms of communication. However, participants were happy to be contacted a week before for data collection measures, a day before data collection measures, and to be reminded three times to complete. If no reply was received after three contact points, then it was suggested that the participant is no longer interested. General updates about the study, with a reminder as to how their data is helping, what information the team has gained thus far, and what is next, were happy to be received every few months. A further reminder of the benefits to participants was suggested by participants not in education.

Participants placed importance on those working with them in data collection. Discussions detailed that the person taking measurements should be ‘approachable’, and ‘non-judgemental’. They preferred consistency with who was collecting data from them, and who was communicating with them. A Sixth Former discussion detailed a preference for ‘talking to someone you trust’. Another participant stated:

“I’d rather build a relationship with the person first before I talk to them.”

## Discussion

The current study explored adolescents’ perspectives of signing up to and continuing involvement in a hypothetical longitudinal research study, with a secondary focus on population health. Adolescents and young adults indicated that strategies for recruitment and retention in research should be engaging, feature those of a similar age, come from a trusted institution, employ simple messaging, and reflect the need to adapt recruitment messages to focus on specific age groups. Participants favoured recruitment via social media, preferred financial incentives, joining with peer groups, and user friendly and flexible data collection. An overarching concept from the results is social connection. Participants valued connection with peers, and the research team. This concept permeates personalised feedback, a desire for a relationship before data collection, peer involvement, and the importance of non-judgemental and consistent staff in supportive data collection.

In the current study, participants discussed being more positive and motivated to sign-up and participate in research when joining with peers. This finding is in line with results from previous studies in this patient population. Recruitment of teens by teens [42], in particular, from a friend [43], and the use of snowball sampling strategies among adolescents have been successful methods for increasing recruitment [15]. There are various rationales for this behaviour. LaRose et al., [21] found that peer support and having someone who was going through a similar journey were important factors linked to recruitment and retention. Peers may also provide a vetting process to involvement in the research. Involvement in research may be unknown, or may be intimidating [44]. Social norms are suggested to influence the initiation and maintenance of a variety of behaviours [45–47]. If peers can normalise engagement with research and break down perceived barriers to participation, this could provide an ‘in’ to recruitment and facilitate ongoing engagement. This links to the theory of social facilitation, or Introjection, where there is an increase in performance (or, in this case, sign up), as a result of the presence of others [48], or where participants are motivated by obligation/guilt or a desire for approval from others [24]. In addition, involvement of peers in behavioural health intervention efforts has been shown to be critical for promoting and maintaining positive trajectories [49]. Capitalizing on this approach, research has moved to incentivised peer recruitment or referral [15, 27].

Establishing a relationship with researchers was identified as an important consideration for involvement in research. Building trusting relationships between research staff and participants helps to personalise participants’ involvement, and make data collection less intimidating [50]. Discussions around the consistency of research staff, and the importance of non-judgemental research staff, with good interpersonal skills are consistent with other studies that have reported these as vital to successful retention in longitudinal studies [51]. While it can be difficult to build a relationship ahead of an in-person visit, the use of social media may provide opportunities to establish relationships, prior to seeing someone in person, which could feel especially clinical. Researchers/teams may potentially learn from the relationships that brands and influencers develop with their followers.

Results show a desire for a 20–30-min monthly data collection process, the provision of flexible scheduling, app-based data collection, the avoidance of overburdening communication, and good interpersonal skills and consistency of the data collection team. Previous research has indicated that adolescents express a preference toward technology-based assessment methods [52]. App-based data collection is documented as having the possibility to reduce participant burden [53], and also opens possibilities for new approaches, for example, for delivering and collecting information, facilitating communication between patients and professionals, social networking, capturing real-time data, monitoring bodily functions, automated feedback, guidance and clinical alerts, and smart decision-making tools [48]. Given that adolescents readily accept and adopt new technologies [54], employing available technology that is incorporated easily into their lifestyle may help with study retention. Additionally, communicating via an app has the capacity to ameliorate some concerns about keeping up to date with participant contact details if participants retain the app. Although email engagement has shown to be successful at retaining adolescents over a 2-year period [19], future app-focussed research should look to review app notifications as a follow-up engagement method to note its effectiveness. Noteworthy, as found in previous research, longitudinal studies need to find a balance between overburdening participants and providing enough personal contact to retain participants [55].

In contrast to previous evidence, being informed how their data helped others was not the most discussed factor for engaging with research. In a previous study, 43% of adolescent participants who met the diagnostic criteria for moderate to severe depression suggested that helping others and contributing to wider understanding was their main motivation for participating in a randomised clinical trial [56]. Similarly, Saarijärvi et al., [57] found adolescents with more complex conditions felt grateful towards research and the healthcare system and were thus motivated to ‘give back’. A population with a specific common interest (e.g., a medical condition) could potentially have more altruistic motivations. Participants in the current study wanted to understand the wider purpose of the study and were interested in the research aims more broadly. Their focus was on about them feeling valued as a participant and their desire for personalised feedback.

The results presented in this study emphasize incentives as an important motivator for participant sign-up, and the offer of incentives for participation were assumed from this age group. Participants preferred continued financial incentives, along with personalised feedback. Payment is common practice for previous research with adolescent samples [58], and incentives have the capacity to maximise representativeness and increase participation [59, 60]. Additionally, offering incentives to participants throughout the research process can provide an ‘immediate tangible benefit’ to participants [61], further enhancing motivations to continued participation.

A central question concerns how to motivate young people to participate in the research process when interest is low. Understanding motivations for participation may play an important role in answering this question. A sub-theory of SDT, Organismic Integration Theory (OIT), details different forms of extrinsic motivation and the contextual factors that either promote or hinder internalization and integration of the regulation for behaviour [62]. Informed by the findings of this study, Table 2 illustrates an adapted OIT taxonomy of types of motivation, arranged from left to right in terms of the extent to which the motivation for one’s behaviour emanates from oneself, and details shows how this process can be applied to recruitment and retention.

**Table 2:** Extrinsic motivation continuum applied to research study recruitment – adapted from Ryan and Deci [42]

Regulatory styles	External Regulation	Introjection	Identification	Integration
**Associated processes**	Motivated by reward or avoiding negative outcome	Motivated by obligation/guilt or a desire for approval from others	Motivated by an appreciation for the activity and importance of achieving goals	Motivated by an alignment between the goals of an activity with other personal goals and values
**Example**	Taking part in a physical activity study to receive a £10 gift voucher	Taking part in a study on physical activity as friends have already signed-up	Taking part in a study because improving physical activity is an important personal goal	Taking part in a study because it aligns with broader beliefs and values; there are no internal conflicts or incongruities
**Perceived local of causality**	External	Somewhat external	Somewhat internal	Internal

Odierna and Bero’s [26] focus group study suggests that participant motivations for enrolling in a study can serve either as a barrier or facilitator. Motivations serve as a barrier if participants’ original motivations (e.g., need for information on rare diseases, money, etc.) for joining the study are not met. Motivations can serve as a facilitator if motivations are adequately addressed or altruistic in nature (e.g., belief in research, volunteerism). By understanding the process of external motivation alongside common motivations for participating in the research process, researchers may be able to better understand the perceived benefits of research participation from young people’s perspectives. The current study shows that participants want to feel respected, valued as an individual and want to learn about themselves, while also feeling socially connected. Understanding adolescent and young adult motivations to sign-up and continue in research may differ from current explanations with other populations [63, 64], and requires further research. Noteworthy, few empirical studies have examined the role of participant motivations for enrolling in a study or directly related these motivations to retention. Price et al., [55], identified demographic factors and having a positive experience in the study to date as primary reasons for continued involvement in a study.

### Strengths and limitations

The strengths of this study include the qualitative nature of the design and the purposeful sampling to specifically include a range of different subgroups. The focus group format and convenience and snowball sampling allowed us to generate rich information not easily found using other research designs [65]. The contribution to both in school/college recruitment and non-school/college recruitment is a key strength of this study. Moreover, demographic information helped to situate the sample [66]. This research contributes to the limited qualitative studies in the field, and provides an update to previous qualitative literature [25].

Despite being a primary focus of the study, recruitment for young adults NEET was a particular challenge. Recruitment approaches included meetings with local charities (e.g., a rotary club, and one that focussed on people NEET), emails with the local council and various local employers but ultimately snowball sampling was the only effective recruitment method. Future studies may also want to allocate budget to test the viability of social media recruitment for this target population.

The recruitment of participants, in particular, the school selection of eligible participants may have impacted the results of the study. The sample was less ethnically diverse and included a larger proportion of females when compared with UK Census data. [67]. It is likely that more diverse perspectives would have been shared by other subgroups, including participants from low socioeconomic backgrounds, or more ethnically diverse groups. Engagement outside of formal research, such as through public involvement or co-design may be more suitable to gain the views of harder to reach population on recruitment and retention strategies for specific research projects. In addition, the current sample is community-based, and not a sample of research participants. Whilst their perceptions will provide transferable insights, there may be differences between a specifically recruited clinical sample with existing health conditions.

### Future research

While the results are based on adolescent participants’ perspectives of participating in a hypothetical longitudinal health study, researchers may be able to apply the results and conclusions to a wider range of studies. Combining the findings from the current study with the wider literature on adolescent recruitment and retention, we identify the following recommendations for future research including adolescent participation:

- Researchers need to understand their target population [17], along with their motivations and interest. This will affect the communication of the research e.g., if participants are not interested in physical activity, then the framing of the research needs to be mindful of this, as well as their continued involvement e.g., in understanding what is important to their continued involvement, e.g., the provision of feedback.
- Invest in extensive Patient and Public Involvement (PPI) and testing of strategies prior to recruitment, and throughout. The recruitment and retention medium needs to be contemporary and align with the target group. We note that this is difficult with this population if recruitment is spread over several years e.g., relevance of social media platforms changes, and as such, needs to be kept up to date. Additionally, allocation of a budget to test the viability of recruitment and retention strategies, for example, social media recruitment methods, need to be considered.
- Health promotion interventions should incorporate an understanding of adolescents’ maturation, concerns and priorities [68]. The research protocol should be sensitive to adolescent development, autonomy, milestone events, and key transitional periods to maximize its success in both recruitment and retention. Tailoring of protocols and interventions to appeal to young people moving through transitions should be designed to appeal to and meet their unique needs [21]. Flexibility, choice, and autonomy may allow participants to feel involved in the research process and take ownership over their continued involvement in research over this transitional period of adolescents into early adulthood.
- Work to establish relationships before the study ‘starts.’ Social media, and/or an app should be considered as a mechanism used to promote connectedness within the research.
- Consider the importance of an investment in long-term study staff, with focused training on being non-judgemental.
- The research protocol for longitudinal studies should detail a planned, systematic approach of participant follow-up and detail the effectiveness of their recruitment and retention strategies. A recent systematic review highlighted ‘poor’ reporting of recruitment retention information across 107 behavioural interventions targeting nutrition and physical activity [69]. Additional detail should be provided in reports on the success of each method, e.g., how many participants signed-up; how many participants returned/continued involvement in the study, and after what period. Failure to track refusals to continue involvement in a study could be considered a limitation as future recruitment and retention strategies can be developed to address these issues [70]. Additionally, this insight could develop researcher’s awareness around motivations for participation and engagement.

## Conclusion

Recruitment and retention of adolescents transitioning into early adulthood presents many challenges. This study highlights the importance of understanding young people’s motivations behind study enrolment or dropout, to allow a tailored approach to recruitment and retention in longitudinal health research. Varied modes of recruitment, informed by ongoing public involvement, should be used to successfully recruit, and retain adolescents and young adults. Financial incentives and joining with peer groups were driving factors for sign-up, whilst user-friendly data collection, mixing online (apps) and in-person data collection with non-judgemental and approachable research staff, alongside the provision of flexible scheduling, were identified as key to continued involvement. It is imperative for researchers embarking on research with adolescents to consider the transitional nature of the adolescence, the motivations for participation of this population and to recognise participants as individuals to better promote engagement in research.

## Supplementary Information

**Additional file 1.**

## Data Availability

The data cannot be made openly available because of ethical and legal considerations. Non-identifiable data can be made available to bona-fide researchers on submission of a reasonable request to datasharing@mrc-epid.cam.ac.uk The principles and processes for accessing and sharing data are outlined in the MRC Epidemiology Unit Data Access & Data Sharing Policy

## Abbreviations

GoActive: Get others Active
NEET: Not in education, employment, or training
FE: Further Education
