# Supplementary material for: Recruitment and retention into longitudinal health research from an adolescent perspective: a qualitative study
Source: BMC Med Res Methodol. 2023 Jan 16;23:16. doi: 10.1186/s12874-022-01802-7 (PMC9841671; doi:10.1186/s12874-022-01802-7)
Supplement: Supplementary file 1 — Additional file 1. [file 12874_2022_1802_MOESM1_ESM.pdf]

Topic guide for FAIA focus groups

Project: "Following adolescents into adulthood (FAIA): A qualitative study to investigate how to recruit and retain adolescents and young adults in longitudinal cohort studies".

NB: This topic guide will be adjusted to reflect different participant groups (i.e., school students, working adults). This is a flexible topic guide; the order and content of questions will vary according to the flow of the discussion; and additional prompts may be added.

- Group welcome, introductions
- Explain what a focus group is
- Explain the aim of the focus group, check informed consent (including right to withdraw) and explain confidentiality
- Check understanding of long-term study (give participants an understanding of what could be involved)

Recruitment

1. What would be the best way to approach you about participating in a long-term study?
    - Prompt: social media
2. What would encourage you to participate in a long-term study measuring lifestyle behaviours such as diet and physical activity, and health?
    - Prompt: Why would you take part?
    - Prompt: Would anything stop you from signing up?
3. What would you want to know before the study starts?
4. What concerns would you have about the study?

Data collection (e.g., practicalities)

5. What would be the easiest way for you to answer the questions that we have?
    - Prompt: To collect data, would you prefer to attend a visit, complete a questionnaire by post or use a smartphone or computer?
    - Prompt: How would you feel about installing an app on your phone for data collection?
6. If we posted you an activity monitor, what would encourage you to wear it?
7. When is the best time for you to take part?
    - Prompt: think about the time of year, time of day, and during the week
8. How long would you be willing to spend on data collection?
    - Prompt: Would you prefer short, frequent data collection (several times in a week or even day) or one longer session?
9. If the study could provide you with incentives, what incentives would you be interested in?
    - Prompt: smaller individual incentive or a larger prize draw?

Retention

10. How would you like to be kept in touch with throughout a long-term study?
    - Prompt: social media, app based, letters, emails etc.
11. How would you feel about giving us a wide range of contact details for us to keep in touch with you?

12. How could we keep you interested in participating?
    - Probe: What incentives would you be interested in for follow-up visits? E.g., feedback from data collection, financial incentives etc. If money, how much would keep you interested in participating?
13. How many times is it acceptable for us to contact you?

For post school participants

- Thinking back, what would have kept you participating in a long-term study from the age of 16 until now?
- Thinking back, what would have stopped you from participating in a long-term research study?

Overall thoughts

- Do you have any final thoughts you would like to add?
- Do you have any questions to ask me?
